# Comparison of six anthropometric measures in discriminating diabetes: A cross‐sectional study from the National Health and Nutrition Examination Survey

**DOI:** 10.1111/1753-0407.13295

**Published:** 2022-07-16

**Authors:** Xiao‐cong Liu, Ying‐shan Liu, Hai‐xia Guan, Ying‐qing Feng, Jian Kuang

**Affiliations:** ^1^ Department of Cardiology, Guangdong Cardiovascular Institute, Guangdong Provincial People's Hospital Guangdong Academy of Medical Sciences Guangzhou China; ^2^ Department of Endocrinology, Guangdong Provincial People's Hospital Guangdong Academy of Medical Sciences Guangzhou China; ^3^ The Second School of Clinical Medicine Southern Medical University Guangzhou China

**Keywords:** anthropometry, conicity index, diabetes mellitus, obesity, waist‐to‐height ratio, 人体测量学, 锥度指数, 糖尿病, 肥胖, 腰高比

## Abstract

**Background:**

Traditional anthropometric measures, including body mass index (BMI), are insufficient for evaluating the risk of diabetes. This study aimed to evaluate the performance of new anthropometric measures and a combination of anthropometric measures for identifying diabetes.

**Methods:**

A total of 46 979 participants in the National Health and Nutrition Examination Survey program were included in this study. Anthropometric measures, including weight, BMI, waist circumference (WC), waist‐to‐height ratio (WtHR), conicity index (CI), and A Body Shape Index (ABSI), were calculated. Logistic regression analysis and restricted cubic splines were used to evaluate the association between the anthropometric indices and diabetes. The receiver operating characteristic (ROC) curve analysis was performed to compare the discrimination of different anthropometric measures.

**Results:**

All anthropometric measures were positively and independently associated with the risk of diabetes. After adjusting for covariates, the per SD increment in WC, WtHR, and CI increased the risk of diabetes by 81%, 83%, and 81%, respectively. In the ROC analysis, CI showed superior discriminative ability for diabetes (area under the curve 0.714), and its optimum cutoff value was 1.31. Results of the combined use of BMI and other anthropometric measures showed that among participants with BMI <30 kg/m^2^, an elevated level of another metric increased the risk of having diabetes (*P* < .001). Similarly, at low levels of weight, CI, and ABSI, an elevated BMI increased diabetes risk (*P* < .001).

**Conclusions:**

WtHR and CI had the best ability to identify diabetes when applied to the US noninstitutionalized population. Anthropometric measures containing WC information could improve the discrimination ability.

## Graphical abstract

**Highlights**

- Our study retrospectively analyzed 46 979 members of the noninstitutionalized US population and found that anthropometric parameters, including weight, body mass index, waist circumference (WC), waist‐to‐height ratio (WtHR), A Body Shape Index, and conicity index (CI), had a significant positive correlation with diabetes.
- CI, a novel central‐obesity index based on WC, weight, and height, showed the strongest discriminatory power among these six indices.
- WtHR is the second‐best anthropometric index after CI; with the advantages of being robust and straightforward, WtHR may be a good alternative for identifying diabetes.

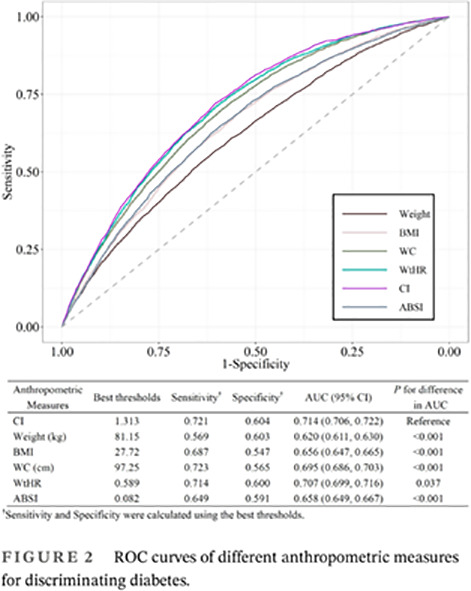

## 摘要

**目的:**

包括体重指数(BMI)在内的传统人体测量方法不足以评估糖尿病的风险。这项研究旨在评估新的人体测量方法和人体测量方法的组合在识别糖尿病方面的表现。

**材料和方法:**

本研究共纳入46979名参加国家健康与营养调查项目的受试者。计算人体测量指标，包括体重、BMI、腰围(WC)、腰高比(WtHR)、圆锥度指数(CI)和身体形态指数(ABSI)。使用Logistic回归分析和限制三次样条法评估人体测量指标与糖尿病之间的关系。对受试者工作特征(ROC)曲线进行分析，比较不同人体测量指标的识别率。

**结果:**

所有人体测量指标均与糖尿病风险呈正相关且独立相关。校正协变量后，WC、WTHR和CI的每标准差增量分别使糖尿病风险增加81%、83%和81%。在ROC分析中，CI对糖尿病具有较好的鉴别能力(曲线下面积为0.714)，其最佳临界值为1.31。体重指数和其它人体测量指标的联合使用结果显示，在BMI<30kg/m^2^的参与者中，另一项指标的升高增加了患糖尿病的风险(P<0.001)。同样，体重、CI和ABSI值较低时，BMI升高会增加患糖尿病的风险(P<0.001)。

**结论:**

在美国非住院人群中，WtHR和CI识别糖尿病的能力最强。包含WC信息的人体测量指标可以提高辨别能力。

## 1 INTRODUCTION

Diabetes mellitus and its complications pose a rapidly growing global health care burden.^1^ Diabetes is associated with various metabolic and cardiovascular risk factors that contribute to high cardiovascular disease (CVD) morbidity and mortality.^2^ Excessive adiposity accumulation, particularly visceral fat deposition, is an established risk factor.^3^

The pathogenesis of obesity‐induced diabetes is complex and multifactorial. Anthropometric measures can provide a convenient and effective prescreening tool for identifying individuals with obesity because they are noninvasive, cost‐effective, valid, and easy to implement. The body mass index (BMI) is the most common measure of general obesity. However, increasing evidence has proven that BMI cannot sufficiently represent the actual obesity status since it cannot accurately reflect body composition and fat location.^4^ Although abdominal fat has been considered when measuring waist circumference (WC), which is the most commonly applied measure of central obesity, it still ignores the effect of height. Consequently, some studies have proposed the waist‐to‐height ratio (WtHR) as a better index for identifying cardiometabolic risk.^5^, ^6^ Nevertheless, various studies conducted in different populations have pointed out that the WtHR is not always an optimal parameter.^7^, ^8^ However, comparisons among these anthropometric indices have not provided sufficient information for any of them to have an absolute advantage in predicting diabetes.^9^

Over the past few decades, several novel anthropometric indices have been proposed to estimate obesity, particularly central obesity. In 2012, a new composite anthropometric index, the A Body Shape Index (ABSI), calculated by normalizing WC to weight and height, was developed by Krakauer et al and appeared to be a satisfactory predictor of mortality independent of BMI.^10^ Conicity index (CI) is another composite index based on WC, weight, and height.^11^ One study showed that CI could better discriminate 10‐year cardiovascular events than WC and WtHR.^12^ However, less is known about the performance of these two indices in identifying the risk of diabetes. Determining the optimal anthropometric index for diabetes screening remains a challenge.

This study aimed to determine and compare the discriminatory performance of six anthropometric indices (weight, BMI, WC, WtHR, ABSI, and CI) as instruments for screening diabetes risk in a large multiracial cohort. The optimal cutoff values of these indices were also defined to help health care professionals and health policy makers assess diabetes risk; subsequently, risk‐reducing interventions could be targeted at participants at high risk, thus reducing the risk of developing diabetes.

## 2 MATERIALS AND METHODS

### 2.1 Study design and participants

This study included data from the 1999/2000 to 2017/2018 cycles of the National Health and Nutrition Examination Survey (NHANES). The NHANES is a multistage, cross‐sectional, and probability sample survey research program designed to obtain a representative sample of the noninstitutionalized US population. The methods used in this study are described in detail in http://www.cdc.gov/nchs/nhanes.htm. The study procedures were approved by the Institutional Review Board of the Centers for Disease Control and Prevention, and all participants provided written informed consent. We included all participants aged ≥18 years. The exclusion criteria included pregnancy and missing anthropometric data. In addition, as the effects of glucose‐lowering therapy may affect the participants' body weight, individuals taking oral hypoglycemic agents or insulin were also excluded from the main analysis. Therefore, the final sample size of the main analysis was 46 979 individuals (Figure **S1**).

### 2.2 Anthropometric measures

Anthropometric data were collected by trained health technicians at the Mobile Examination Center (MEC).^13^ All participants were weighed in kilograms using a digital weight scale and wore a standard MEC examination gown during the examination. WC was measured immediately above the uppermost lateral border of the right ilium of the pelvis. BMI was calculated as weight (kg) divided by the square of height (m). The WtHR was calculated as WC (cm) divided by height (cm). CI and ABSI were calculated according to published formulae^10^, ^11^ as followed:

$$\mathrm{CI}=\frac{{\mathrm{WC}}_{\left(m\right)}}{\sqrt{\frac{{\text{Weight}}_{\left(\mathrm{kg}\right)}}{{\text{Height}}_{\left(m\right)}}}}$$

$$\text{ABSI}\;=\;WC_{\left(m\right)}÷\left(\mathrm{BM}I^{\frac{2}{3}}\times \text{Heigh}{t^{\frac{1}{2}}}_{\left(m\right)}\right)$$

Given the statistical independence of ABSI and BMI (as indicated in subsequent results), the Anthropometric risk index (ARI) was computed as followed:

$$Log\left(ARI\right)=\log\left(OR_{BMI}\right)+\log\left(OR_{ABSI}\right)$$

### 2.3 Outcomes

Diabetes was the dependent variable in this study. The criteria for diagnosis were based on the American Diabetes Association Standards of Medical Care in Diabetes 2018.^15^ Participants meeting one or more of the following criteria were considered to have diabetes: plasma fasting glucose ≥7.0 mmol/L, glycosylated hemoglobin ≥6.5%, self‐reported diabetes diagnosis, or currently taking hypoglycemic agents or insulin.

### 2.4 Covariates

Demographic characteristics and medical history were obtained using a structured questionnaire. Age (years) was used as a continuous variable. Sex was classified as male or female. Race was classified as Mexican American, non‐Hispanic White, non‐Hispanic Black, other Hispanic, and others. Smoking status was categorized as yes or no. Education was divided into two levels: less than high school and high school or above. Marital status was categorized into married and other groups. Blood pressure measures were repeated three times after resting quietly for 5 minutes, and the average value was recorded. The estimated glomerular filtration rate (eGFR) was calculated according to the Chronic Kidney Disease Epidemiology Collaboration creatinine equation.^16^ Plasma fasting glucose and glycosylated hemoglobin levels were determined by the assigned laboratory.

### 2.5 Statistical methods

For descriptive statistics, continuous variables are presented as mean with SD, and categorical variables are described as counts with percentages. Pearson's chi‐square test, independent *t* tests, and Mann‐Whitney *U* test were performed to compare the differences between participants with and without diabetes. The association between the different anthropometric measures and diabetes was estimated using univariate and multivariate logistic regression analyses. All anthropometric variables were z‐standardized when treated as continuous variables. In multivariate logistic regression analysis, two sets of models were established, among which covariates were selected according to clinical importance: model I, adjusted for age, sex, and race, and model II, further adjusted for study cycle, smoking, educational level, marital status, physical activity, systolic blood pressure, diastolic blood pressure, eGFR, and hypertension. We used restricted cubic splines with three knots (10th, 50th, and 90th of exposure) to evaluate the nonlinear association between different anthropometric measures and diabetes, and the median value of each anthropometric measure was used as a reference. Odds ratios (ORs) were logarithm‐transformed to represent the rate of increase. The distribution of the variables is also presented using histograms. Subgroup analyses were performed to explore the effect of heterogeneity. Receiver operating characteristic (ROC) curves and area under the curve (AUC) were used to compare the discriminative power of different anthropometric indices to identify participants with diabetes. Delong's test was used to assess statistical differences between the AUCs. We further examined whether integrating BMI with other anthropometric measures could better assess the risk of diabetes. BMI and WC were dichotomized according to the World Health Organization (WHO) guidelines^17^; obesity was defined as BMI ≥ 30 kg/m^2^, whereas abdominal obesity was defined as WC ≥ 102 cm in men or WC ≥ 88 cm in women. Other anthropometric indices were dichotomized according to the cutoff point in the ROC analysis. Correlation analysis between the two anthropometric indices was performed using Person's correlation analysis. Sensitivity analyses were conducted including diabetes patients with glucose‐lowering therapy. The sample size in sensitivity analyses was 51 438. All data in this study were analyzed using R software (R Core Team, 2020; version 4.0.3). Two‐sided *P* values <.05 were considered statistically significant.

## 3 RESULTS

### 3.1 Baseline characteristics

The study sample consisted of 46 979 individuals, of whom 3517 (7.49%) had diabetes. The participants were 49.8% male and 43.6% non‐Hispanic White, with a mean age of 46.15 years. Demographic and clinical data at baseline are shown in Table 1. Overall, participants with diabetes were older (58.1 vs 45.2 years), more likely to be male (52.4% vs 49.6%), had a higher proportion of smokers (50.5% vs 43.1%) and married (54.1% vs 47.5%), and had lower educational levels (63.0% vs 73.8%) (all *P* < .01). Compared with participants without diabetes, eGFR (87.7 vs 98.7 mg/min/1.73m^2^) were significantly lower, and systolic blood pressure (132.2 vs 122.7 mm Hg) was significantly higher in participants with diabetes (all *P* < .001). In addition, all six anthropometric parameters (weight, BMI, WC, WtHR, CI, and ABSI) involved in this study were significantly elevated in participants with diabetes (all *P* < .001). The baseline characteristics grouped by sex are shown in Table **S1**. Men presented a significantly higher weight (85.1 vs 74.2 kg), WC (98.6 vs 94.8 cm), CI (1.30 vs 1.29), and ABSI (0.081 vs 0.080) compared to women (*P* < .001). In contrast, BMI (27.9 vs 28.7 kg/m^2^) and WtHR (0.57 vs 0.59) were significantly higher in women than in men (*P* < .001).

**TABLE 1:** Baseline characteristics

Variables	Total	Non‐diabetes	Diabetes	*P* value
Number	46 979	43 462	3517	
Age, y	46.15 ± 18.91	45.18 ± 18.83	58.06 ± 15.62	<.001***
Sex‐male, n (%)	23 391 (49.8)	21 548 (49.6)	1843 (52.4)	.001**
Race, n (%)				<.001***
Mexican American	8532 (18.2)	7788 (17.9)	744 (21.2)	
Other Hispanic	3825 (8.1)	3499 (8.1)	326 (9.3)	
Non‐Hispanic White	20 473 (43.6)	19 191 (44.2)	1282 (36.5)	
Non‐Hispanic Black	9876 (21.0)	8988 (20.7)	888 (25.2)	
Other	4273 (9.1)	3996 (9.2)	277 (7.9)	
Smoking, n (%)	19 840 (43.6)	18 078 (43.1)	1762 (50.5)	<.001***
Education level‐high school or above, n (%)	34 295 (73.0)	32 080 (73.8)	2215 (63.0)	<.001***
Married, n (%)	22 546 (48.0)	20 642 (47.5)	1904 (54.1)	<.001***
Physical activity, n (%)				<.001***
Less than moderate	21 621 (46.0)	19 476 (44.8)	2145 (61.0)	
Moderate activity	12 033 (25.6)	11 138 (25.6)	895 (25.4)	
Vigorous activity	13 325 (28.4)	12 848 (29.6)	477 (13.6)	
Hypertension, n (%)	17 042 (36.3)	14 798 (34.0)	2244 (63.8)	<.001***
Systolic blood pressure, mm Hg	123.44 ± 18.85	122.73 ± 18.50	132.22 ± 20.80	<.001***
Diastolic blood pressure, mm Hg	70.28 ± 12.97	70.25 ± 12.81	70.60 ± 14.83	.133
eGFR, mg/min/1.73m^2^	97.82 ± 24.15	98.66 ± 23.87	87.68 ± 25.23	<.001***
Plasma fasting glucose, mmol/L	5.68 ± 1.46	5.41 ± 0.56	8.39 ± 3.47	<.001***
Glycosylated hemoglobin, %	5.51 ± 0.74	5.38 ± 0.38	6.97 ± 1.75	<.001***
Anthropometric measures				
Weight, kg	79.61 ± 20.50	78.94 ± 20.14	87.99 ± 22.88	<.001***
BMI, kg/m^2^	28.30 ± 6.54	28.02 ± 6.39	31.66 ± 7.32	<.001***
WC, cm	96.68 ± 15.96	95.86 ± 15.67	106.88 ± 16.01	<.001***
WtHR	0.58 ± 0.10	0.57 ± 0.09	0.64 ± 0.10	<.001***
CI	1.29 ± 0.09	1.29 ± 0.09	1.36 ± 0.08	<.001***
ABSI	0.081 ± 0.005	0.081 ± 0.005	0.084 ± 0.005	<.001***

*Note*: Values are mean with SD or number with percent.

Abbreviations: ABSI, A Body Shape Index; BMI, body mass index; CI, conicity index; eGFR, estimated glomerular filtration rate; WC, waist circumference; WtHR, waist‐to‐height ratio.

*** *P* value < .001; ** *P* value <.01.

### 3.2 Associations between different anthropometric measures and diabetes

**FIGURE 1:**
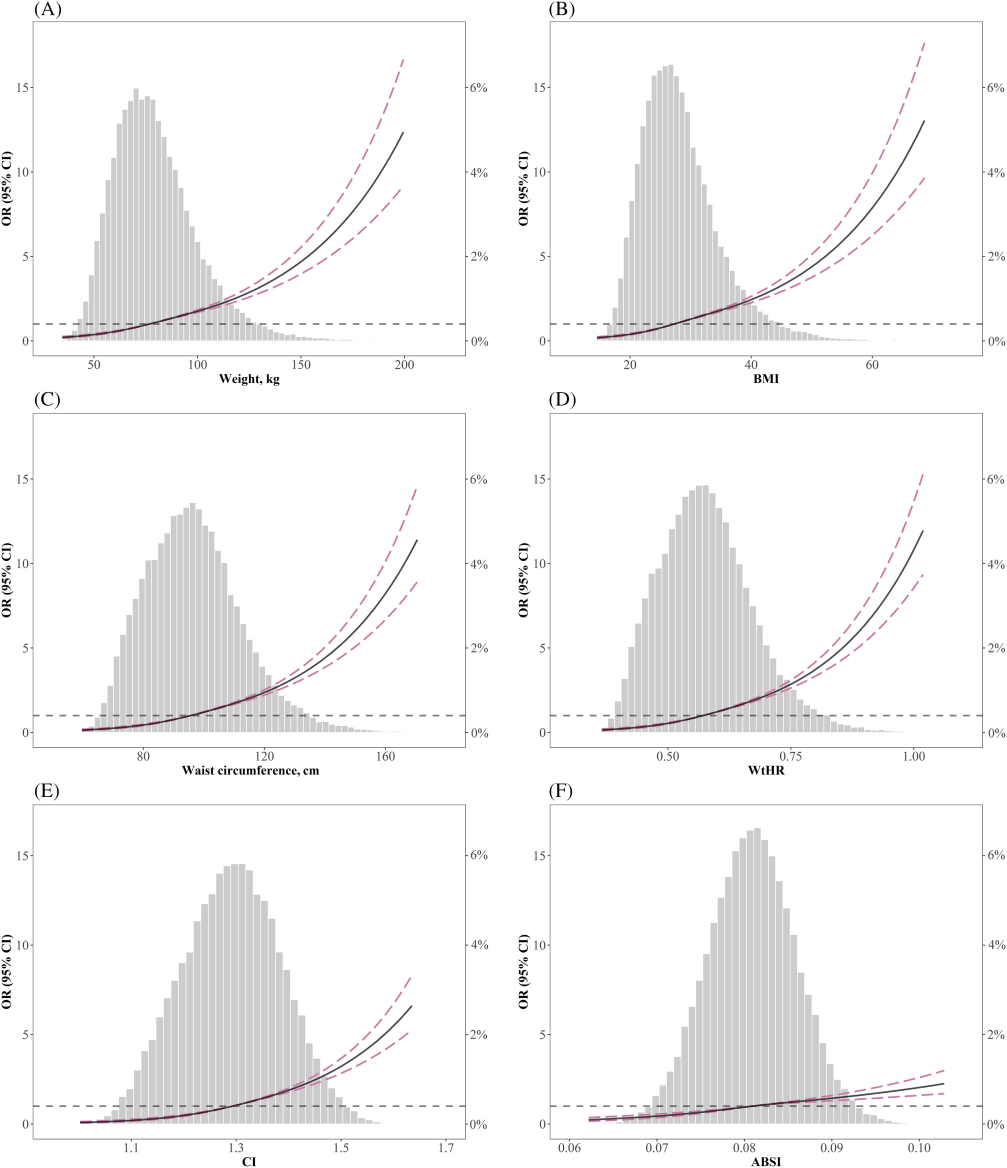
Spline analyses of diabetes and participants' anthropometric measures, with the probability distribution histogram is represented in the background (Spline analyses were adjusted for age, sex, race, study cycle, smoking, education, marriage status, physical activity, systolic blood pressure, diastolic blood pressure, eGFR, and hypertension).

The distribution of the six anthropometric measures is shown in Figure 1. All anthropometric measures had a positive association with diabetes, irrespective of whether adjustment was made for confounding factors (Table 2). In univariate logistic regression analysis, CI had the highest OR per SD change among the six anthropometric measures (OR 2.19; 95% confidence interval, 2.11‐2.28; *P* < .001). After adjustment in multivariate logistic analysis, WC (OR 1.81; 95% confidence interval, 1.74‐1.88; *P* < .001), WtHR (OR 1.83; 95% confidence interval, 1.76‐1.90; *P* < .001), and CI (OR 1.81; 95% confidence interval, 1.72‐1.89; *P* < .001) were associated with the highest OR per SD increment. Moreover, restricted cubic spline analysis was performed to evaluate whether nonlinear associations existed between anthropometric measures and diabetes. As presented in Figure 1, weight, BMI, WC, WtHR, CI, and ABSI were positively associated with diabetes in a nonlinear pattern. The risks of diabetes displayed a relatively rapid increase in the upper quantile of these parameters.

**TABLE 2:** Logistic regression analysis of anthropometric measures and diabetes

	Nonadjusted		Model I		Model II	
	OR (95% confidence interval)	*P* value	OR (95% confidence interval)	*P* value	OR (95% confidence interval)	*P* value
Weight	1.46 (1.42, 1.50)	<.001***	1.69 (1.64, 1.75)	<.001***	1.65 (1.59, 1.71)	<.001***
BMI	1.57 (1.53, 1.62)	<.001***	1.70 (1.64, 1.75)	<.001***	1.65 (1.59, 1.71)	<.001***
WC	1.86 (1.80, 1.92)	<.001***	1.86 (1.80, 1.93)	<.001***	1.81 (1.74, 1.88)	<.001***
WtHR	1.93 (1.87, 1.99)	<.001***	1.89 (1.82, 1.96)	<.001***	1.83 (1.76, 1.90)	<.001***
CI	2.19 (2.11, 2.28)	<.001***	1.88 (1.80, 1.96)	<.001***	1.81 (1.72, 1.89)	<.001***
ABSI	1.72 (1.66, 1.78)	<.001***	1.33 (1.27, 1.39)	<.001***	1.29 (1.23, 1.35)	<.001***
ARI (ABSI, BMI)	2.02 (1.94, 2.12)	<.001***	1.83 (1.73, 1.93)	<.001***	1.75 (1.65, 1.86)	<.001***

*Note*: Data are ORs, 95% confidence intervals, and *P* values for per SD increment. Model I adjusted for age, sex, and race. Model II adjusted for age, sex, race, study cycle, smoking, education, marital status, physical activity, systolic blood pressure, diastolic blood pressure, eGFR, and hypertension.

Abbreviations: ABSI, A Body Shape Index; ARI, anthropometric risk index; BMI, body mass index; CI, conicity index; eGFR, estimated glomerular filtration rate; OR, odds ratio; WC, waist circumference; WtHR, waist‐to‐height ratio.

*** *P* value < .001.

In the subgroup analysis (Table **S2**), participants were separated by age (≥60 and <60 years), sex (male and female), BMI (≥30 and <30 kg/m^2^), and race (White, Black, and others). The results showed that most anthropometric measures were more strongly associated with diabetes in younger individuals (*P*_interaction_ < .01, except for BMI, WC, and WtHR) and those with a BMI less than 30 kg/m^2^ (all *P*_interaction_ < .001). Compared with men, the increases in WC were more highly correlated with diabetes in women. (*P*_interaction_ < .05). The non‐Hispanic White population was more sensitive to traditional anthropometric measures (weight, BMI, and WtHR) than the non‐Hispanic Black and other populations (all *P*_interaction_ < .05, *P*_interaction_ for WC also close to significance). However, there was no interaction effect between race and CI (*P*_interaction_ >.05).

### 3.3 Discrimination ability of different anthropometric measures

**FIGURE 2:**
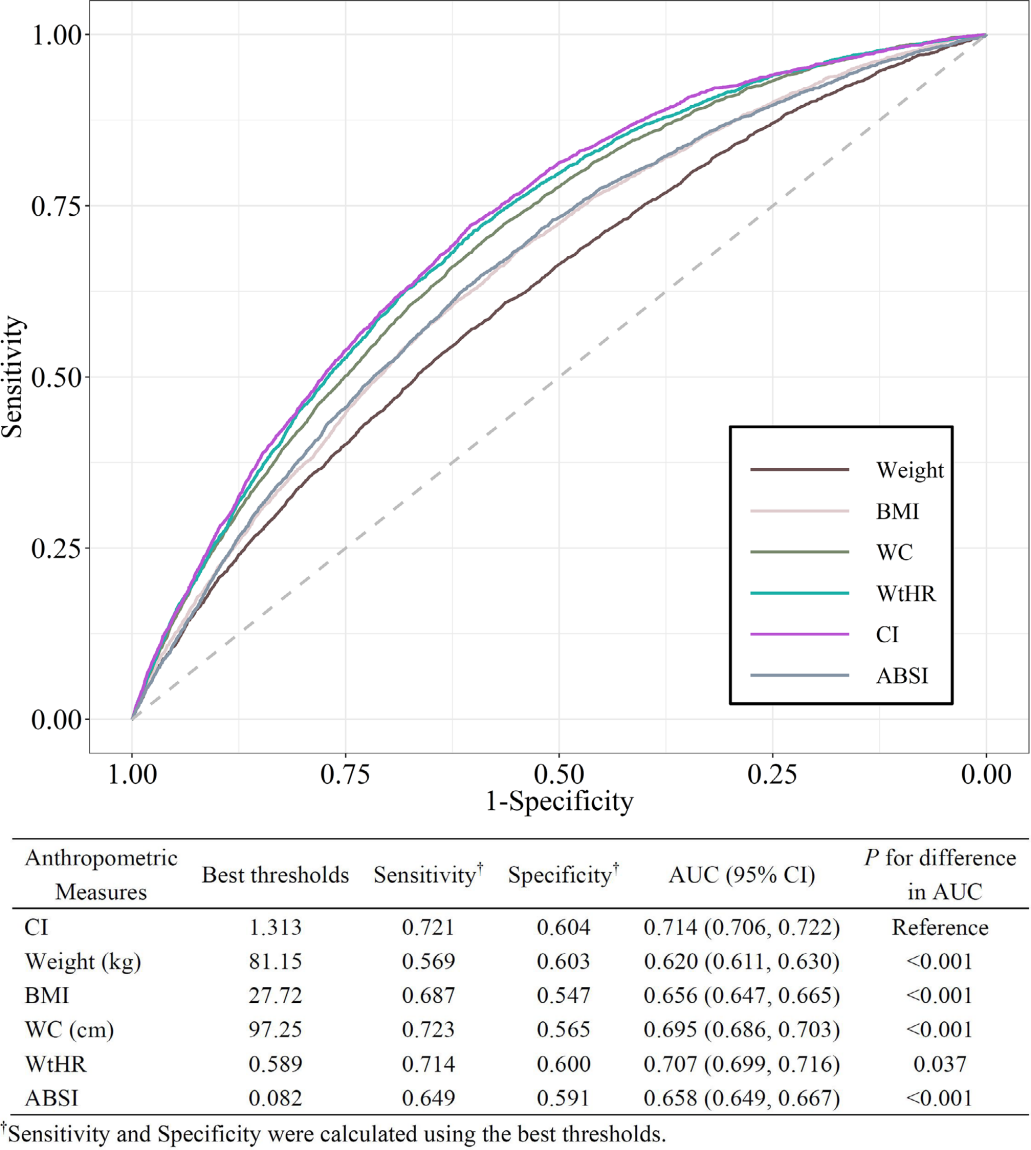
ROC curves of different anthropometric measures for discriminating diabetes.

The ROC curves for identifying participants with diabetes are shown in Figure 2. CI showed the best discrimination ability (AUC 0.714; 95% confidence interval, 0.706‐0.722) among all six anthropometric parameters (Delong's test all *P* < .05). The optimal cutoff value of CI with the highest Youden index was 1.31, which had a sensitivity of 0.72 and a specificity of 0.60. Moreover, the AUCs for weight, BMI, WC, WtHR, and ABSI were 0.620 (95% confidence interval, 0.611‐0.630), 0.656 (95% confidence interval, 0.647‐0.665), 0.695 (95% confidence interval, 0.686‐0.703), 0.707 (95% confidence interval, 0.699‐0.716), and 0.658 (95% confidence interval, 0.649‐0.667), respectively. Separate ROC curve analyses were also performed on the subgroups of participants with and without a BMI ≥ 30 kg/m^2^ (Table **S3**). Similarly, CI exhibited the best discrimination power for both subgroups. In addition, all anthropometric measures showed higher AUC values in participants without obesity.

### 3.4 Combination of BMI and other anthropometric indices

**FIGURE 3:**
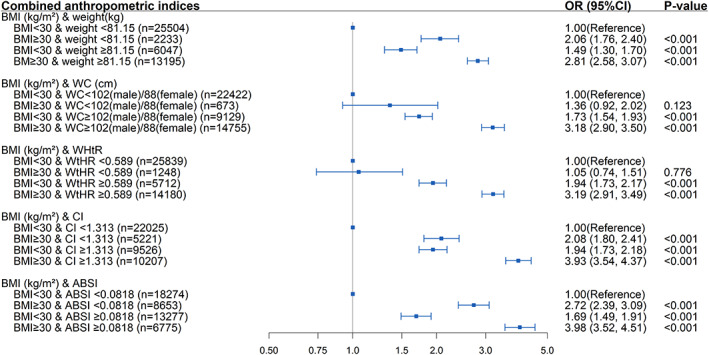
Association between diabetes and combined anthropometric indices. (Adjusted for age, sex, race, study cycle, smoking, education, marriage status, physical activity, systolic blood pressure, diastolic blood pressure, eGFR, and hypertension).

The correlation analysis of the different anthropometric measures is shown in Table **S4**. The correlation with BMI was the strongest for WtHR (r = 0.91) and moderate for CI (r = 0.50). ABSI was negligibly correlated with BMI (r = 0.01). As shown in Figure 3, in participants with a BMI < 30 kg/m^2^, elevated weight (OR 1.49; 95% confidence interval, 1.30‐1.70; *P* < .001), WC (OR 1.73; 95% confidence interval, 1.54‐1.93; *P* < .001), WtHR (OR 1.94; 95% confidence interval, 1.73‐2.17; *P* < .001), CI (OR 1.94, 95% confidence interval, 1.73‐2.18; *P* < .001), and ABSI (OR 1.69; 95% confidence interval, 1.49‐1.91; *P* < .001) were all positively correlated with diabetes. Similarly, in participants with normal weight, CI, and ASBI, an elevated BMI increased diabetes risk (*P* < .001). However, participants with a BMI ≥ 30 kg/m^2^ but normal WC or WtHR did not have an increased risk of diabetes (both *P* > .05). Additionally, participants with a BMI ≥ 30 kg/m^2^ along with other abnormal anthropometric measures were associated with the highest risk of diabetes.

### 3.5 Sensitivity analysis

As shown in Table **S5**, anthropometric measures were significantly lower in participants without diabetes than in those with diabetes, regardless of medication use. The sensitivity analyses were consistent with the main analyses. After including participants receiving glucose‐lowering therapy, WC, WtHR, and CI still had higher ORs among the six anthropometric measures (Table **S6**). CI still had the best discrimination ability among the six anthropometric parameters (Figure S2; AUC 0.743; Delong's test all *P* < .05), and the optimal cutoff points with the highest Youden index were also close to the main results.

## 4 DISCUSSION

The present large cross‐sectional study revealed that all six anthropometric indices examined (weight, BMI, WC, WtHR, ABSI, and CI) were positively correlated with diabetes risk, supporting that weight and WC reduction will reduce the population incidence of diabetes. Among these six indices, CI showed the strongest discriminatory power. WtHR has a similarly discriminative ability next to CI with a simpler calculation.

Obesity is an established risk factor for diabetes.^18^ In this study, four central‐obesity indices (WC, WtHR, ABSI, and CI) had superior discrimination ability for screening diabetes risk compared to the indices of general obesity (weight and BMI), reconfirming that central obesity is more strongly associated with diabetes risk than general obesity.^19^ Our findings also indicated that individuals with a normal BMI but central obesity had an elevated risk of diabetes. BMI is an index measuring both fat and fat‐free mass and may not adequately represent abdominal fat distribution. Individuals with normal weight but central obesity have excessive abdominal fat. Their normal BMI suggests that these individuals are at risk for decreased muscle mass and bone density compared to those with the same BMI and no central obesity.^20^ Previous studies have confirmed that excessive visceral fat deposition in patients with central obesity exacerbates insulin resistance and β‐cell damage due to the higher inflammatory activity in abdominal visceral fat,^21^, ^22^ resulting in impaired glucose tolerance and eventually prompting the diabetes development.^23^, ^24^ Conversely, muscle mass and bone density manifest a strong connection with a more favorable metabolic status, and decreased muscle mass and bone density can potentially result in reduced protective roles with adverse health outcomes.^25^, ^26^, ^27^ Therefore, measuring the indices of central obesity in addition to BMI can provide incremental benefits in the prescreening of diabetes.

Although central‐obesity indices are generally considered better than BMI in screening for diabetes, controversy still exists regarding which central‐obesity measures perform the best.^19^ In the present study, the AUC value of WtHR for diabetes screening was close to that of WC as expected from the correlation between WC and WtHR given in Table S4 as 0.93. The advantage of the WtHR lies in the comprehensive consideration of height and WC, which is less affected by race, age, and sex, and thus is relatively stable. However, correlation analysis among different anthropometric measures showed a strong correlation between WtHR and BMI, indicating that WtHR may not be an effective complementary indicator of BMI. In addition, it is worth noting that at least eight WC measurement locations were reported in previous studies. Among them, two WC measurement locations, WC‐mid (measuring WC in the horizontal plane midway between the lowest ribs and iliac crest) and WC‐IC (measured in the horizontal plane of the superior border of the iliac crest), are the most representative because they were recommended by the WHO, International Diabetes Federation, and National Cholesterol Education Programme‐Adult Treatment Panel III.^28^, ^29^, ^30^ Ma et al compared WC‐mid and WC‐IC, revealing that the performance of WC‐IC and WC‐mid in identifying hypertension, diabetes, and metabolic syndrome was similar. However, WC‐mid proved to be a better measurement to define central obesity than WC‐IC and had better results for predicting metabolic diseases in Asians.^31^ The WC‐IC data were used in the NHANES data, which might limit the comparability of the present study's results with WC measured in various anatomical locations. Because other central‐obesity indices involved in this study were all calculated using a simple linear formula based on WC‐IC, we hypothesize that these central‐obesity indices might have better screening effectiveness if better location measurements, such as WC‐mid, are used in specific ethnic groups. However, this did not affect the ranking among the different indices because the human waist is consistent; thus, the values of different WC measurement locations are correlated. We are therefore confident in the conclusion of the present study. Meanwhile, we suggest that the location of WC measurement should be considered before comparing the effectiveness and optimal cutoff values of the different indices.

We further analyzed CI and ABSI, which incorporate height and weight with WC. The basic formula is given by WC × Height^*x*^/Weight^*y*^. For CI, *x* = 0.5 and *y* = 0.5, while for ABSI, *x* = 5/6 and *y* = 2/3, whereas the correlations with BMI are 0.50 and 0.01, respectively. We found that CI performed significantly better in screening diabetes than other indices, regardless of whether the individuals had obesity or not. Previous studies have found that CI was superior to BMI for identifying visceral adiposity,^32^ metabolic disorders, and cardiovascular risk factors.^33^ Thus, CI was proposed as an effective instrument to determine the risk of CVD. At present, the use of CI in screening for diabetes has been less studied. Neufeld conducted a small cross‐sectional study of young Mexican women and found that the best cutoff point of CI for prediabetes was 1.28 for CI.^34^ This is consistent with our findings from NHANES with a CI cutoff of 1.31.

ABSI shows a lower OR in screening for diabetes compared to other central‐obesity indices. However, ABSI was negligibly correlated with BMI, establishing statistical independence and therefore applicability of ABSI to the present cohort. This finding implies that after correction for BMI, the association of WC with DM incidence is attenuated, findings that were compatible with a Chinese study of insulin resistance.^35^ Similar findings have been reported in a review comparing body roundness index with ABSI for predicting hypertension.^36^ Certainly, ABSI and CI are cousin power laws that differ only in terms of their exponents and constants. ABSI by design was originally derived from NHANES to capture the variation in WC that is not dependent on BMI, whereas CI is confounded by BMI. We found better discrimination when ABSI was combined with BMI. This might be interpreted as the fact that changes in ABSI do not entirely reflect the real metabolism on their own since MS, diabetes, and hypertension are all multifactorial disorders. Therefore, future studies are warranted to determine whether ABSI can be used in combination with BMI to screen for diabetes. Krakauer et al suggested that the ARI, a novel risk indicator that utilizes complementary information from height, hip index, BMI, and ABSI, is a substantially better predictor of mortality risk than any of the individual anthropometric indices tested.^14^, ^37^ The present study also demonstrates the ARI to be a potential favorable predictive index, which performed better than BMI and ABSI alone.

This study has several merits and implications. First, our study was based on a large‐sample multicenter study; the sample size was adequate to provide sufficient statistical power. Second, we used restricted cubic splines to reveal the nonlinear associations between different measures and diabetes and intuitively showed the OR for different levels of obesity. Third, the central‐obesity indices based on WC have better discriminatory power for screening diabetes than BMI. It is strongly recommended that WC should be part of a participant's medical history in addition to BMI. Fourth, from the point of view of public health, using CI as a noninvasive, simple screening tool could offer a practical approach for screening the risk of diabetes. However, our study has some limitations. First, this was a cross‐sectional study; thus, causal associations could not be determined. Second, the fat content and distribution data were unavailable in this study. Therefore, we cannot directly assess the association of anthropometric measures with body fat and visceral fat. Third, the NHANES data were not able to distinguish between type 1 and 2 diabetes. Fourth, some variables in this study were self‐reported and may have experienced subjective bias. Fifth, the NHANES study was based on the US noninstitutionalized population. Consequently, the results of this study may not be applicable to other populations.

## 5 CONCLUSION

In this study, we retrospectively analyzed 46 979 members of the US noninstitutionalized population and found that anthropometric parameters, including weight, BMI, WC, WtHR, ABSI, and CI, had a significant positive correlation with diabetes. Measuring BMI alone is insufficient for assessing the risk of diabetes. The CI and WtHR are the strongest discriminators of diabetes and may be used as good indicators for screening diabetes.

## DISCLOSURE

The authors have no conflicts of interest to disclose.

## Supporting information


**Figure S1**. Study flowchart.
**Figure S2.** Sensitivity analysis of different anthropometric measures for discriminating diabetes (Including diabetes patients with glucose‐lowering therapy, *n* = 51 438).
**Table S1.** Baseline characteristics grouped by sex
**Table S2.** Subgroups analysis
**Table S3.** ROC analyses in BMI subgroup
**Table S4.** Pearson correlation analysis among different Anthropometric Measures.
**Table S5.** Comparison of baseline characteristics among participants without diabetes, diabetes patients without any treatments, and diabetes patients with glucose‐lowering therapy.
**Table S6.** Sensitivity analysis of Anthropometric Measures and Diabetes (Including diabetes patients with glucose‐lowering therapy, *n* = 51 438).

## References

[1] Ogurtsova K , da Rocha Fernandes JD , Huang Y , et al. IDF diabetes atlas: global estimates for the prevalence of diabetes for 2015 and 2040. Diabetes Res Clin Pract. 2017;128:40‐50. 28437734 10.1016/j.diabres.2017.03.024

[2] Hunt KJ , Jaffa MA , Garrett SM , et al. Plasma connective tissue growth factor (CTGF/CCN2) levels predict myocardial infarction in the veterans affairs diabetes trial (VADT) cohort. Diabetes Care. 2018;41(4):840‐846. 29382658 10.2337/dc17-2083 PMC5860844

[3] Meshram II , Vishnu Vardhana Rao M , Sudershan Rao V , Laxmaiah A , Polasa K . Regional variation in the prevalence of overweight/obesity, hypertension and diabetes and their correlates among the adult rural population in India. Br J Nutr. 2016;115(7):1265‐1272. 26867590 10.1017/S0007114516000039

[4] Heymsfield SB , Scherzer R , Pietrobelli A , Lewis CE , Grunfeld C . Body mass index as a phenotypic expression of adiposity: quantitative contribution of muscularity in a population‐based sample. Int J Obes (Lond). 2009;33(12):1363‐1373. 19773739 10.1038/ijo.2009.184 PMC3156622

[5] Ashwell M , Gunn P , Gibson S . Waist‐to‐height ratio is a better screening tool than waist circumference and BMI for adult cardiometabolic risk factors: systematic review and meta‐analysis. Obes Rev. 2012;13(3):275‐286. 22106927 10.1111/j.1467-789X.2011.00952.x

[6] Lo K , Liu Q , Allison M , et al. Prospective associations of waist‐to‐height ratio with cardiovascular events in postmenopausal women: results from the Women's Health Initiative. Diabetes Care. 2019;42(9):e148‐e149. 31308018 10.2337/dc19-0612 PMC6702600

[7] Janssen I , Katzmarzyk PT , Ross R . Waist circumference and not body mass index explains obesity‐related health risk. Am J Clin Nutr. 2004;79(3):379‐384. 14985210 10.1093/ajcn/79.3.379

[8] Menke A , Muntner P , Wildman RP , Reynolds K , He J . Measures of adiposity and cardiovascular disease risk factors. Obesity (Silver Spring). 2007;15(3):785‐795. 17372330 10.1038/oby.2007.593

[9] van Dijk SB , Takken T , Prinsen EC , Wittink H . Different anthropometric adiposity measures and their association with cardiovascular disease risk factors: a meta‐analysis. Neth Heart J. 2012;20(5):208‐218. 22231153 10.1007/s12471-011-0237-7 PMC3346869

[10] Krakauer NY , Krakauer JC . A new body shape index predicts mortality hazard independently of body mass index. PLoS One. 2012;7(7):e39504. 22815707 10.1371/journal.pone.0039504 PMC3399847

[11] Valdez R . A simple model‐based index of abdominal adiposity. J Clin Epidemiol. 1991;44(9):955‐956. 1890438 10.1016/0895-4356(91)90059-i

[12] Motamed N , Perumal D , Zamani F , et al. Conicity index and waist‐to‐hip ratio are superior obesity indices in predicting 10‐year cardiovascular risk among men and women. Clin Cardiol. 2015;38(9):527‐534. 26418518 10.1002/clc.22437 PMC6490781

[13] Centers for Disease Control and Prevention . NHANES ‐ National Health and Nutrition Examination Survey Homepage (2020). https://www.cdc.gov/nchs/nhanes/index.htm. Accessed March 15, 2020.

[14] Krakauer NY , Krakauer JC . An anthropometric risk index based on combining height, weight, waist, and hip measurements. J Obes. 2016;2016:8094275. 27830087 10.1155/2016/8094275 PMC5088335

[15] Chamberlain JJ , Johnson EL , Leal S , Rhinehart AS , Shubrook JH , Peterson L . Cardiovascular disease and risk management: review of the American Diabetes Association standards of medical Care in Diabetes 2018. Ann Intern Med. 2018;168(9):640‐650. 29610837 10.7326/M18-0222

[16] Inker LA , Schmid CH , Tighiouart H , et al. Estimating glomerular filtration rate from serum creatinine and cystatin C. N Engl J Med. 2012;367(1):20‐29. 22762315 10.1056/NEJMoa1114248 PMC4398023

[17] World Health Organization . Waist circumference and waist‐hip ratio: report of a WHO expert consultation, Geneva, 8–11 December 2008. https://www.who.int/nutrition/publications/obesity/WHO_report_waistcircumference_and_waisthip_ratio/en/. Accessed May 12, 2021.

[18] Cosentino F , Grant PJ , Aboyans V , et al. 2019 ESC guidelines on diabetes, pre‐diabetes, and cardiovascular diseases developed in collaboration with the EASD. Eur Heart J. 2020;41(2):255‐323. 31497854 10.1093/eurheartj/ehz486

[19] Neeland IJ , Ross R , Després J‐P , et al. Visceral and ectopic fat, atherosclerosis, and cardiometabolic disease: a position statement. Lancet Diabetes Endocrinol. 2019;7(9):715‐725. 31301983 10.1016/S2213-8587(19)30084-1

[20] Ludescher B , Machann J , Eschweiler GW , et al. Correlation of fat distribution in whole body MRI with generally used anthropometric data. Invest Radiol. 2009;44(11):712‐719. 19809346 10.1097/RLI.0b013e3181afbb1e

[21] Piche ME , Tchernof A , Despres JP . Obesity phenotypes, diabetes, and cardiovascular diseases. Circ Res. 2020;126(11):1477‐1500. 32437302 10.1161/CIRCRESAHA.120.316101

[22] Stumvoll M , Goldstein BJ , van Haeften TW . Type 2 diabetes: principles of pathogenesis and therapy. Lancet. 2005;365(9467):1333‐1346. 15823385 10.1016/S0140-6736(05)61032-X

[23] Piche ME , Poirier P , Lemieux I , Despres JP . Overview of epidemiology and contribution of obesity and body fat distribution to cardiovascular disease: an update. Prog Cardiovasc Dis. 2018;61(2):103‐113. 29964067 10.1016/j.pcad.2018.06.004

[24] Fox CS , Massaro JM , Hoffmann U , et al. Abdominal visceral and subcutaneous adipose tissue compartments: association with metabolic risk factors in the Framingham heart study. Circulation. 2007;116(1):39‐48. 17576866 10.1161/CIRCULATIONAHA.106.675355

[25] Srikanthan P , Karlamangla AS . Muscle mass index as a predictor of longevity in older adults. Am J Med. 2014;127(6):547‐553. 24561114 10.1016/j.amjmed.2014.02.007 PMC4035379

[26] Srikanthan P , Horwich TB , Tseng CH . Relation of muscle mass and fat mass to cardiovascular disease mortality. Am J Cardiol. 2016;117(8):1355‐1360. 26949037 10.1016/j.amjcard.2016.01.033

[27] Li R , Xia J , Zhang XI , et al. Associations of muscle mass and strength with all‐cause mortality among US older adults. Med Sci Sports Exerc. 2018;50(3):458‐467. 28991040 10.1249/MSS.0000000000001448 PMC5820209

[28] Alberti KG , Zimmet PZ . Definition, diagnosis and classification of diabetes mellitus and its complications. Part 1: diagnosis and classification of diabetes mellitus provisional report of a WHO consultation. Diabet Med. 1998;15(7):539‐553. 9686693 10.1002/(SICI)1096-9136(199807)15:7<539::AID-DIA668>3.0.CO;2-S

[29] Grundy SM , Cleeman JI , Daniels SR , et al. Diagnosis and management of the metabolic syndrome: an American Heart Association/National Heart, Lung, and Blood Institute scientific statement. Circulation. 2005;112(17):2735‐2752. 16157765 10.1161/CIRCULATIONAHA.105.169404

[30] Alberti KG , Zimmet P , Shaw J . Metabolic syndrome–a new world‐wide definition. A Consensus Statement from the International Diabetes Federation. Diabet Med. 2006;23(5):469‐480. 16681555 10.1111/j.1464-5491.2006.01858.x

[31] Ma WY , Yang CY , Shih SR , et al. Measurement of waist circumference: midabdominal or iliac crest? Diabetes Care. 2013;36(6):1660‐1666. 23275359 10.2337/dc12-1452 PMC3661855

[32] Andrade MD , Freitas MC , Sakumoto AM , et al. Association of the conicity index with diabetes and hypertension in Brazilian women. Arch Endocrinol Metab. 2016;60(5):436‐442. 27812606 10.1590/2359-3997000000187 PMC10118640

[33] Tarastchuk JC , Guérios EE , Bueno Rda R , et al. Obesity and coronary intervention: should we continue to use body mass index as a risk factor? Arq Bras Cardiol. 2008;90(5):284‐289. 18516395 10.1590/s0066-782x2008000500001

[34] Neufeld LM , Jones‐Smith JC , García R , Fernald LC . Anthropometric predictors for the risk of chronic disease in non‐diabetic, non‐hypertensive young Mexican women. Public Health Nutr. 2008;11(2):159‐167. 17601359 10.1017/S136898000700002X

[35] Wu K , He S , Zheng Y , Chen X . ABSI is a poor predictor of insulin resistance in Chinese adults and elderly without diabetes. Arch Endocrinol Metab. 2018;62(5):523‐529. 30462805 10.20945/2359-3997000000072 PMC10118652

[36] Calderón‐García JF , Roncero‐Martín R , Rico‐Martín S , et al. Effectiveness of body roundness index (BRI) and a body shape index (ABSI) in predicting hypertension: a systematic review and meta‐analysis of observational studies. Int J Environ Res Public Health. 2021;18(21):11607. 10.3390/ijerph182111607 PMC8582804 34770120

[37] Krakauer NY , Krakauer JC . Untangling waist circumference and hip circumference from body mass index with a body shape index, hip index, and anthropometric risk indicator. Metab Syndr Relat Disord. 2018;16(4):160‐165. 29649376 10.1089/met.2017.0166

