# Supplementary material for: Comparison of six anthropometric measures in discriminating diabetes: A cross‐sectional study from the National Health and Nutrition Examination Survey
Source: J Diabetes. 2022 Jul 16;14(7):465–75. doi: 10.1111/1753-0407.13295 (PMC9310044; doi:10.1111/1753-0407.13295)
Supplement: Supplementary file 1 — Figure S1. Study flowchart. Figure S2. Sensitivity analysis of different anthropometric measures for discriminating diabetes (Including diabetes patients with glucose‐lowering therapy, n = 51 438). Table S1. Baseline characteristics grouped by sex Table S2. Subgroups analysis Table S3. ROC analyses in BMI subgroup Table S4. Pearson correlation analysis among different Anthropometric Measures. Table S5. Comparison of baseline characteristics among participants without diabetes, diabetes patients without any treatments, and diabetes patients with glucose‐lowering therapy. Table S6. Sensitivity analysis of Anthropometric Measures and Diabetes (Including diabetes patients with glucose‐lowering therapy, n = 51 438). [file JDB-14-465-s001.docx]

**Table S1** Baseline characteristics grouped by sex

| Variables | Male | | | | Female | | | | P-value between  male and female |
| --- | --- | --- | --- | --- | --- | --- | --- | --- | --- |
|  | Total | Non-diabetes | Diabetes | P-value | Total | Non-diabetes | Diabetes | P-value |  |
| Number | 23391 | 21548 | 1843 |  | 23588 | 21914 | 1674 |  |  |
| Age, years | 45.80±19.00 | 44.74±18.90 | 58.13±15.51 | <0.001^***^ | 46.50±18.82 | 45.62±18.75 | 57.97±15.76 | <0.001^***^ | <0.001^***^ |
| Race, n (%) |  |  |  | <0.001^***^ |  |  |  | <0.001^***^ | <0.001^***^ |
| Mexican American | 4294 (18.4) | 3915 (18.2) | 379 (20.6) |  | 4238 (18.0) | 3873 (17.7) | 365 (21.8) |  |  |
| Other Hispanic | 1744 (7.5) | 1574 (7.3) | 170 (9.2) |  | 2081 (8.8) | 1925 (8.8) | 156 (9.3) |  |  |
| Non-Hispanic White | 10301 (44.0) | 9593 (44.5) | 708 (38.4) |  | 10172 (43.1) | 9598 (43.8) | 574 (34.3) |  |  |
| Non-Hispanic Black | 4910 (21.0) | 4473 (20.8) | 437 (23.7) |  | 4966 (21.1) | 4515 (20.6) | 451 (26.9) |  |  |
| Other | 2142 (9.2) | 1993 (9.2) | 149 (8.1) |  | 2131 (9.0) | 2003 (9.1) | 128 (7.6) |  |  |
| Smoking, n (%) | 11873 (52.5) | 10748 (51.8) | 1125 (61.4) | <0.001^***^ | 7967 (34.8) | 7330 (34.5) | 637 (38.4) | 0.001^**^ | <0.001^***^ |
| Education level- High school or above, n (%) | 16704 (71.4) | 15535 (72.1) | 1169 (63.4) | <0.001^***^ | 17591 (74.6) | 16545 (75.5) | 1046 (62.5) | <0.001^***^ | <0.001^***^ |
| Married, n (%) | 12122 (51.8) | 10972 (50.9) | 1150 (62.4) | <0.001^***^ | 10424 (44.2) | 9670 (44.1) | 754 (45.0) | 0.468 | <0.001^***^ |
| physical activity, n (%) |  |  |  | <0.001^***^ |  |  |  | <0.001^***^ | <0.001^***^ |
| Less than moderate | 10042 (42.9) | 8985 (41.7) | 1057 (57.4) |  | 11579 (49.1) | 10491 (47.9) | 1088 (65.0) |  |  |
| Moderate activity | 5429 (23.2) | 4956 (23.0) | 473 (25.7) |  | 6604 (28.0) | 6182 (28.2) | 422 (25.2) |  |  |
| Vigorous activity | 7920 (33.9) | 7607 (35.3) | 313 (17.0) |  | 5405 (22.9) | 5241 (23.9) | 164 (9.8) |  |  |
| Hypertension, n (%) | 8445 (36.1) | 7319 (34.0) | 1126 (61.1) | <0.001^***^ | 8597 (36.4) | 7479 (34.1) | 1118 (66.8) | <0.001^***^ | 0.440 |
| Systolic blood pressure, mmHg | 124.89±16.89 | 124.33±16.55 | 131.48±19.31 | <0.001^***^ | 121.98±20.52 | 121.14±20.13 | 133.04±22.32 | <0.001^***^ | <0.001^***^ |
| Diastolic blood pressure, mmHg | 71.46±13.26 | 71.42±13.13 | 71.99±14.73 | 0.082 | 69.09±12.56 | 69.09±12.37 | 69.06±14.79 | 0.921 | <0.001^***^ |
| eGFR, mg/min/1.73m^2^ | 96.54±23.24 | 97.43±22.91 | 86.44±24.53 | <0.001^***^ | 99.10±24.96 | 99.86±24.72 | 89.10±25.94 | <0.001^***^ | <0.001^***^ |
| Plasma Fasting Glucose, mmol/L | 5.77±1.49 | 5.48±0.56 | 8.49±3.43 | <0.001^***^ | 5.58±1.42 | 5.33±0.56 | 8.27±3.51 | <0.001^***^ | <0.001^***^ |
| Glycosylated Hemoglobin, % | 5.52±0.77 | 5.39±0.38 | 7.02±1.82 | <0.001^***^ | 5.49±0.70 | 5.38±0.38 | 6.91±1.67 | <0.001^***^ | <0.001^***^ |
| Anthropometric Measures |  |  |  |  |  |  |  |  |  |
| Weight, kg | 85.08±19.61 | 84.49±19.28 | 91.96±22.01 | <0.001^***^ | 74.20±19.91 | 73.48±19.46 | 83.62±23.03 | <0.001^***^ | <0.001^***^ |
| BMI, kg/m^2^ | 27.90±5.74 | 27.67±5.62 | 30.61±6.43 | <0.001^***^ | 28.68±7.22 | 28.37±7.05 | 32.81±8.03 | <0.001^***^ | <0.001^***^ |
| WC, cm | 98.60±15.47 | 97.82±15.19 | 107.72±15.77 | <0.001^***^ | 94.78±16.22 | 93.93±15.90 | 105.95±16.22 | <0.001^***^ | <0.001^***^ |
| WtHR | 0.57±0.09 | 0.56±0.09 | 0.62±0.09 | <0.001^***^ | 0.59±0.10 | 0.58±0.10 | 0.67±0.10 | <0.001^***^ | <0.001^***^ |
| CI | 1.30±0.10 | 1.29±0.09 | 1.36±0.08 | <0.001^***^ | 1.29±0.09 | 1.28±0.09 | 1.35±0.08 | <0.001^***^ | <0.001^***^ |
| ABSI | 0.081±0.005 | 0.081±0.005 | 0.084±0.004 | <0.001^***^ | 0.080±0.005 | 0.082±0.005 | 0.083±0.005 | <0.001^***^ | <0.001^***^ |

Abbreviations: eGFR: estimated glomerular filtration rate; BMI, Body Mass Index; WC, Waist Circumference; WtHR, Waist-to-Height Ratio; CI, Conicity Index; ABSI, A Body Shape Index.

Values are mean with SD or number with percent.

^**^ *P*-value<0.01, ^***^ *P*-value<0.001.

**Table S2** Subgroups analysis

|  | Age | |  | Sex | |  | BMI | |  | Race | | |
| --- | --- | --- | --- | --- | --- | --- | --- | --- | --- | --- | --- | --- |
|  | ≥60 years (n=16148) | <60 years (n=35290) |  | Males (n=25680) | Females (n=25758) |  | ≥30 kg/m^2^ (n=17929) | <30 kg/m^2^ (n=33509) |  | White (n=21995) ^a^ | Black (n=11060) ^b^ | Other (18383) ^c^ |
| Weight | 1.48 (1.40, 1.56) | 1.57 (1.49, 1.64) |  | 1.57 (1.50, 1.65) | 1.68 (1.59, 1.77) |  | 1.37 (1.29, 1.45) | 1.36 (1.27, 1.46) |  | 1.70 (1.60, 1.80) | 1.62 (1.50, 1.74) | 1.59 (1.50, 1.69) |
| P for interaction | 0.008^**^ | |  | 0.055 | |  | <0.001^***^ | |  | 0.044^*^ | 0.243 | 0.259 |
| BMI | 1.48 (1.41, 1.56) | 1.61 (1.54, 1.69) |  | 1.61 (1.53, 1.69) | 1.68 (1.60, 1.77) |  | \ | \ |  | 1.69 (1.60, 1.79) | 1.62 (1.50, 1.75) | 1.61 (1.52, 1.70) |
| P for interaction | 0.084 | |  | 0.112 | |  | \ | |  | 0.006^**^ | 0.044^*^ | 0.363 |
| WC | 1.63 (1.54, 1.72) | 1.81 (1.72, 1.90) |  | 1.72 (1.63, 1.81) | 1.91 (1.81, 2.02) |  | 1.46 (1.39, 1.54) | 1.60 (1.49, 1.71) |  | 1.88 (1.76, 2.00) | 1.82 (1.68, 1.97) | 1.72 (1.62, 1.83) |
| P for interaction | 0.478 | |  | 0.030^*^ | |  | <0.001^***^ | |  | 0.065 | 0.264 | 0.381 |
| WtHR | 1.62 (1.54, 1.71) | 1.86 (1.77, 1.96) |  | 1.74 (1.65, 1.84) | 1.91 (1.80, 2.02) |  | 1.44 (1.37, 1.52) | 1.62 (1.51, 1.73) |  | 1.88 (1.77, 2.00) | 1.84 (1.69, 2.00) | 1.75 (1.65, 1.87) |
| P for interaction | 0.668 | |  | 0.714 | |  | <0.001^***^ | |  | 0.031^*^ | 0.186 | 0.385 |
| CI | 1.55 (1.47, 1.65) | 1.95 (1.84, 2.07) |  | 1.79 (1.67, 1.92) | 1.82 (1.71, 1.94) |  | 1.43 (1.34, 1.52) | 1.60 (1.49, 1.72) |  | 1.83 (1.69, 1.97) | 1.89 (1.72, 2.08) | 1.69 (1.57, 1.81) |
| P for interaction | <0.001^***^ | |  | 0.220 | |  | <0.001^***^ | |  | 0.831 | 0.865 | 0.614 |
| ABSI | 1.22 (1.15, 1.29) | 1.45 (1.37, 1.54) |  | 1.30 (1.21, 1.40) | 1.27 (1.20, 1.35) |  | 1.25 (1.18, 1.33) | 1.41 (1.31, 1.51) |  | 1.25 (1.16, 1.35) | 1.40 (1.27, 1.53) | 1.22 (1.13, 1.31) |
| P for interaction | <0.001^***^ | |  | 0.564 | |  | <0.001^***^ | |  | 0.156 | 0.632 | 0.276 |

When analyzing a subgroup variable age, sex, race, study cycle, smoking, education, marriage status, physical activity, systolic blood pressure, diastolic blood pressure, eGFR, and hypertension were adjusted except for the variable itself.

Abbreviations: BMI, Body Mass Index; WC, Waist Circumference; WtHR, Waist-to-Height Ratio; CI, Conicity Index; ABSI, A Body Shape Index.

^a^ *P* for interaction were calculated by Anthropometric Measures × race (White or Black)

^b^ *P* for interaction were calculated by Anthropometric Measures × race (Black or Other)

^c^ *P* for interaction were calculated by Anthropometric Measures × race (White or Other)

^*^ *P*-value<0.05, ^**^ *P*-value<0.01, ^***^ *P*-value<0.001.

Values are mean with SD or number with percent.

**Table S3** ROC analyses in BMI subgroup

| Subgroups | Anthropometric  Measures | Best thresholds | Sensitivity^†^ | Specificity^†^ | AUC (95% CI) | *P* for difference  in AUC |
| --- | --- | --- | --- | --- | --- | --- |
| BMI≥30 kg/m^2^ |  |  |  |  |  |  |
|  | CI | 1.352 | 0.656 | 0.556 | 0.641 (0.628, 0.654) | Reference |
|  | Weight (kg) | 109.6 | 0.380 | 0.679 | 0.532 (0.517, 0.546) | <0.001^***^ |
|  | WC (cm) | 112.4 | 0.299 | 0.762 | 0.614 (0.6, 0.627) | <0.001^***^ |
|  | WtHR | 0.666 | 0.668 | 0.525 | 0.631 (0.618, 0.644) | 0.091 |
|  | ABSI | 0.082 | 0.586 | 0.600 | 0.620 (0.607, 0.633) | <0.001^***^ |
| BMI<30 kg/m^2^ |  |  |  |  |  |  |
|  | CI | 1.290 | 0.708 | 0.625 | 0.714 (0.701, 0.726) | Reference |
|  | Weight (kg) | 63.85 | 0.752 | 0.341 | 0.557 (0.543, 0.57) | <0.001^***^ |
|  | WC (cm) | 91.45 | 0.673 | 0.594 | 0.679 (0.667, 0.692) | <0.001^***^ |
|  | WtHR | 0.551 | 0.679 | 0.622 | 0.700 (0.687, 0.712) | <0.001^***^ |
|  | ABSI | 0.082 | 0.703 | 0.596 | 0.697 (0.685, 0.71) | <0.001^***^ |

^†^Sensitivity and Specificity were calculated using the best thresholds.

Abbreviations: BMI, Body Mass Index; WC, Waist Circumference; WtHR, Waist-to-Height Ratio; CI, Conicity Index; ABSI, A Body Shape Index.

^***^ *P*-value<0.001.

**Table S4** Pearson correlation analysis among different Anthropometric Measures.

|  | weight | BMI | WC | WtHR | CI | ABSI |
| --- | --- | --- | --- | --- | --- | --- |
| Weight | 1.00 | 0.88 | 0.89 | 0.71 | 0.46 | 0.02 |
| BMI | - | 1.00 | 0.90 | 0.91 | 0.50 | 0.01 |
| WC | - | - | 1.00 | 0.93 | 0.79 | 0.40 |
| WtHR | - | - | - | 1.00 | 0.78 | 0.39 |
| CI | - | - | - | - | 1.00 | 0.87 |
| ABSI | - | - | - | - | - | 1.00 |

Abbreviations: BMI, Body Mass Index; WC, Waist Circumference; WtHR, Waist-to-Height Ratio; CI, Conicity Index; ABSI, A Body Shape Index.

**Table S5** Comparison of baseline characteristics among participants without diabetes, participants with diabetes without hypoglycemic therapy, and participants with hypoglycemic therapy.

| Variables | Non-diabetes | Diabetes patients without treatments | Diabetes patients with glucose-lowing therapy | *P*-value |
| --- | --- | --- | --- | --- |
| Number | 43462 | 3517 | 4459 |  |
| Age, years | 45.18 ± 18.83 | 58.06 ± 15.62 | 62.11 ± 12.85 | <0.001^***^ |
| Sex-male, n (%) | 21548 (49.6) | 1843 (52.4) | 2289 (51.3) | 0.001^**^ |
| Race, n (%) |  |  |  | <0.001^***^ |
| Mexican American | 7788 (17.9) | 744 (21.2) | 885 (19.8) |  |
| Other Hispanic | 3499 (8.1) | 326 (9.3) | 401 (9.0) |  |
| Non-Hispanic White | 19191 (44.2) | 1282 (36.5) | 1522 (34.1) |  |
| Non-Hispanic Black | 8988 (20.7) | 888 (25.2) | 1184 (26.6) |  |
| Other | 3996 (9.2) | 277 (7.9) | 467 (10.5) |  |
| Smoking, n (%) | 18078 (43.1) | 1762 (50.5) | 2222 (49.9) | <0.001^***^ |
| Education level-High school or above, n (%) | 32080 (73.8) | 2215 (63.0) | 2830 (63.5) | <0.001^***^ |
| Married, n (%) | 20642 (47.5) | 1904 (54.1) | 2497 (56.0) | <0.001^***^ |
| physical activity, n (%) |  |  |  | <0.001^***^ |
| Less than moderate | 19476 (44.8) | 2145 (61.0) | 2766 (62.0) |  |
| Moderate activity | 11138 (25.6) | 895 (25.4) | 1269 (28.5) |  |
| Vigorous activity | 12848 (29.6) | 477 (13.6) | 424 (9.5) |  |
| Hypertension, n (%) | 14798 (34.0) | 2244 (63.8) | 3374 (75.7) | <0.001^***^ |
| Systolic blood pressure, mmHg | 122.73 ± 18.50 | 132.22 ± 20.80 | 132.76 ± 20.35 | <0.001^***^ |
| Diastolic blood pressure, mmHg | 70.25 ± 12.81 | 70.60 ± 14.83 | 67.66 ± 14.81 | <0.001^***^ |
| eGFR, mg/min/1.73m^2^ | 98.66 ± 23.87 | 87.68 ± 25.23 | 81.24 ± 26.29 | <0.001^***^ |
| Plasma Fasting Glucose, mmol/L | 5.41 ± 0.56 | 8.39 ± 3.47 | 8.63 ± 3.81 | <0.001^***^ |
| Glycosylated Hemoglobin, % | 5.38 ± 0.38 | 6.97 ± 1.75 | 7.61 ± 1.78 | <0.001^***^ |
| Anthropometric Measures |  |  |  |  |
| Weight, kg | 78.94 ± 20.14 | 87.99 ± 22.88 | 88.88 ± 22.71 | <0.001^***^ |
| BMI, kg/m^2^ | 28.02 ± 6.39 | 31.66 ± 7.32 | 32.14 ± 7.13 | <0.001^***^ |
| WC, cm | 95.86 ± 15.67 | 106.88 ± 16.01 | 109.11 ± 15.99 | <0.001^***^ |
| WtHR | 0.57 ± 0.09 | 0.64 ± 0.10 | 0.66 ± 0.09 | <0.001^***^ |
| CI | 1.29 ± 0.09 | 1.36 ± 0.08 | 1.38 ± 0.08 | <0.001^***^ |
| ABSI | 0.081 ± 0.005 | 0.084 ± 0.005 | 0.084 ± 0.005 | <0.001^***^ |

Abbreviations: eGFR: estimated glomerular filtration rate; BMI, Body Mass Index; WC, Waist Circumference; WtHR, Waist-to-Height Ratio; CI, Conicity Index; ABSI, A Body Shape Index.

*** P-value<0.001.

Values are mean with SD or number with percent.

**Table S****6** Sensitivity analysis of Anthropometric Measures and Diabetes (Including diabetes patients with glucose-lowing therapy, n=51438).

|  | Non-adjusted | | Model I | | Model II | |
| --- | --- | --- | --- | --- | --- | --- |
|  | OR (95% CI) | *P*-value | OR (95% CI) | *P*-value | OR (95% CI) | *P*-value |
| Weight | 1.51 (1.48, 1.55) | <0.001^***^ | 1.86 (1.81, 1.91) | <0.001^***^ | 1.75 (1.70, 1.81) | <0.001^***^ |
| BMI | 1.68 (1.64, 1.71) | <0.001^***^ | 1.87 (1.82, 1.92) | <0.001^***^ | 1.76 (1.71, 1.81) | <0.001^***^ |
| WC | 2.06 (2.01, 2.11) | <0.001^***^ | 2.11 (2.06, 2.17) | <0.001^***^ | 1.98 (1.92, 2.04) | <0.001^***^ |
| WtHR | 2.18 (2.13, 2.24) | <0.001^***^ | 2.14 (2.08, 2.21) | <0.001^***^ | 2.00 (1.94, 2.06) | <0.001^***^ |
| CI | 2.59 (2.52, 2.67) | <0.001^***^ | 2.17 (2.10, 2.24) | <0.001^***^ | 2.00 (1.93, 2.08) | <0.001^***^ |
| ABSI | 1.96 (1.91, 2.01) | <0.001^***^ | 1.44 (1.40, 1.49) | <0.001^***^ | 1.37 (1.33, 1.42) | <0.001^***^ |
| ARI (ABSI, BMI) | 2.34 (2.26, 2.42) | <0.001^***^ | 2.06 (1.98, 2.15) | <0.001^***^ | 1.91 (1.83, 1.99) | <0.001^***^ |

Abbreviations: BMI, Body Mass Index; WC, Waist Circumference; WtHR, Waist-to-Height Ratio; CI, Conicity Index; ABSI, A Body Shape Index; ARI, Anthropometric Risk Index.

Data are odds ratio (OR), 95% confidence intervals (95% CI), and *P*-value for per SD increment.

^***^ *P*-value<0.001.

Model I adjust for age, sex, and race

Model II adjust for age, sex, race, study cycle, smoking, education, marriage status, physical activity, systolic blood pressure, diastolic blood pressure, eGFR, and hypertension.

**
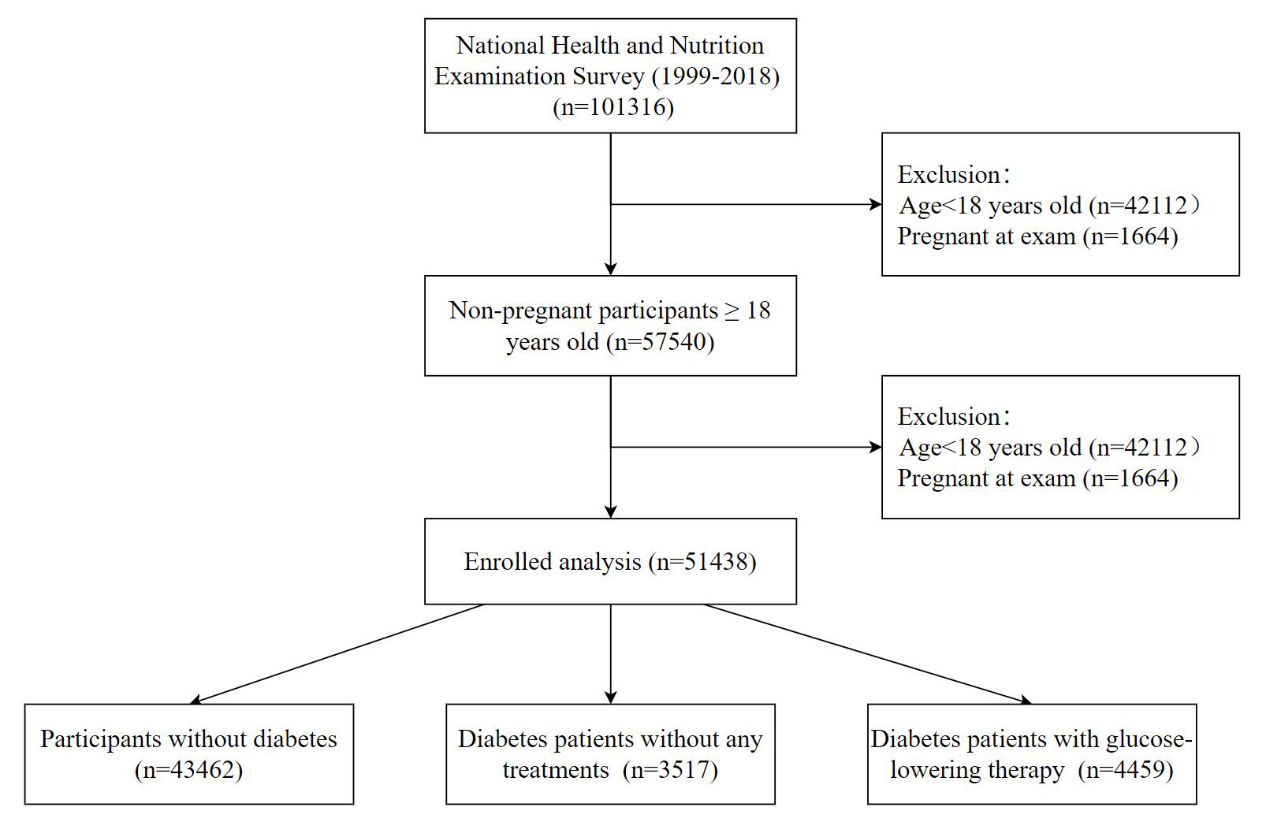
**

Figure S1 Study flowchart.

**
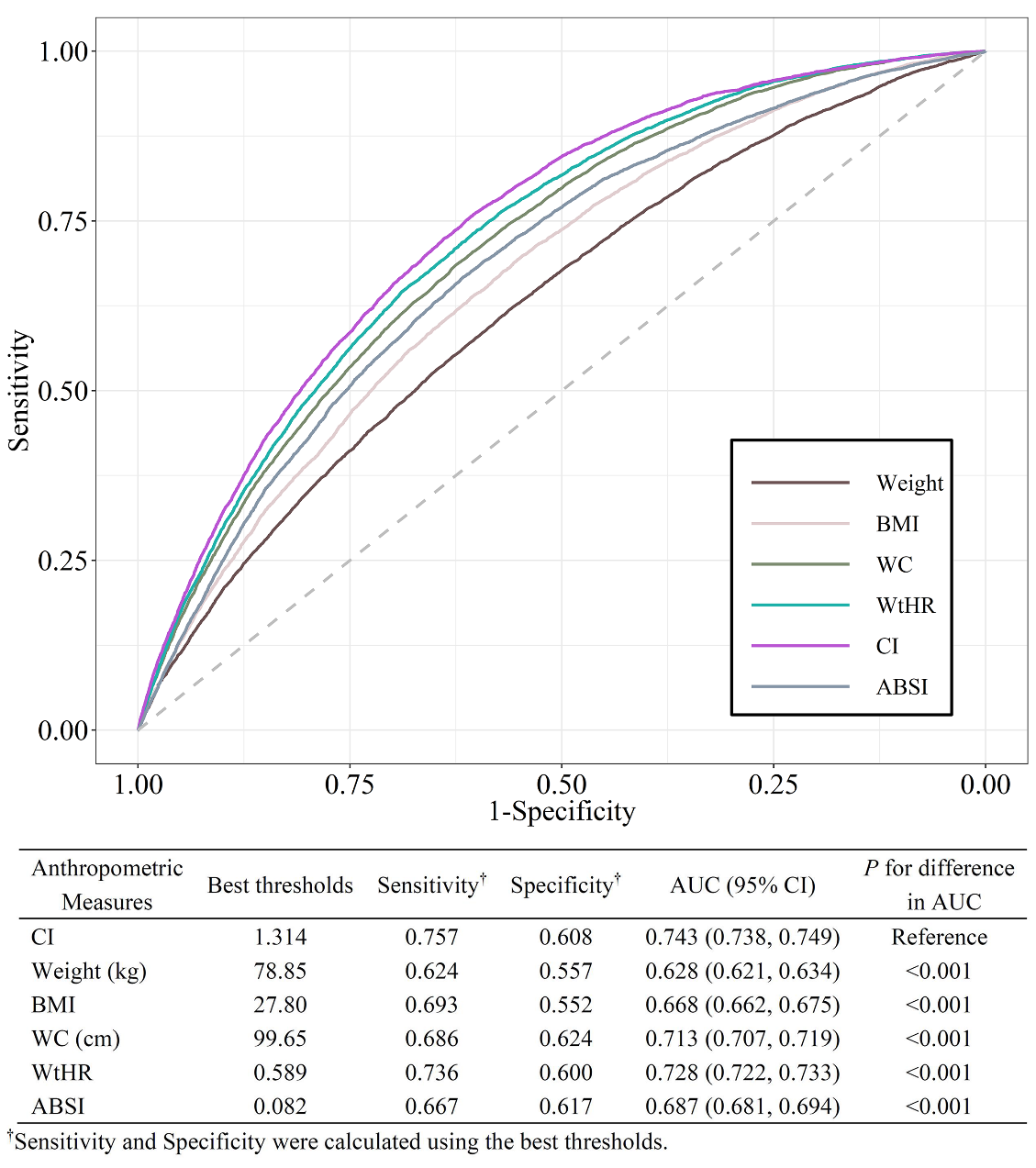
**

**Figure S2** Sensitivity analysis of different anthropometric measures for discriminating diabetes (Including diabetes patients with glucose-lowing therapy, n=51543).
